# Mitochondrial Metabolism behind Region-Specific Resistance to Ischemia-Reperfusion Injury in Gerbil Hippocampus. Role of PKCβII and Phosphate-Activated Glutaminase

**DOI:** 10.3390/ijms22168504

**Published:** 2021-08-07

**Authors:** Małgorzata Beręsewicz-Haller, Olga Krupska, Paweł Bochomulski, Danuta Dudzik, Anita Chęcińska, Wojciech Hilgier, Coral Barbas, Krzysztof Zablocki, Barbara Zablocka

**Affiliations:** 1 Molecular Biology Unit, Mossakowski Medical Research Institute, PAS, 02-106 Warsaw, Poland; mbersewicz@imdik.pan.pl (M.B.-H.); o.krupska@nencki.edu.pl (O.K.); p.bochomulski@gmail.com (P.B.); achecinska@imdik.pan.pl (A.C.); 2 Laboratory of Cellular Metabolism, Nencki Institute of Experimental Biology, PAS, 02-106 Warsaw, Poland; k.zablocki@nencki.gov.pl; 3 Centre for Metabolomics and Bioanalysis (CEMBIO), Department of Chemistry and Biochemistry, Facultad de Farmacia, Universidad San Pablo-CEU, CEU Universities, 28003 Madrid, Spain; danuta.dudzik@gumed.edu.pl (D.D.); cbarbas@ceu.es (C.B.); 4 Department of Biopharmaceutics and Pharmacodynamics, Faculty of Pharmacy, Medical University of Gdańsk, 80-210 Gdańsk, Poland; 5 Department of Neurotoxicology, Mossakowski Medical Research Institute, PAS, 02-106 Warsaw, Poland; whilgier@imdik.pan.pl

**Keywords:** cerebral ischemia, endogenous neuroprotection, mitochondria, glutamate metabolism, metabolomics, protein kinase C, glutaminase 1

## Abstract

Ischemic episodes are a leading cause of death worldwide with limited therapeutic interventions. The current study explored mitochondrial phosphate-activated glutaminase (GLS1) activity modulation by PKCβII through GC-MS untargeted metabolomics approach. Mitochondria were used to elucidate the endogenous resistance of hippocampal CA2-4 and dentate gyrus (DG) to transient ischemia and reperfusion in a model of ischemic episode in gerbils. In the present investigation, male gerbils were subjected to bilateral carotids occlusion for 5 min followed by reperfusion (IR). Gerbils were randomly divided into three groups as vehicle-treated sham control, vehicle-treated IR and PKCβII specific inhibitor peptide βIIV5-3-treated IR. Vehicle or βIIV5-3 (3 mg/kg, i.v.) were administered at the moment of reperfusion. The gerbils hippocampal tissue were isolated at various time of reperfusion and cell lysates or mitochondria were isolated from CA1 and CA2-4,DG hippocampal regions. Recombinant proteins PKCβII and GLS1 were used in in vitro phosphorylation reaction and organotypic hippocampal cultures (OHC) transiently exposed to NMDA (25 μM) to evaluate the inhibition of GLS1 on neuronal viability. PKCβII co-precipitates with GAC (GLS1 isoform) in CA2-4,DG mitochondria and phosphorylates GLS1 in vitro. Cell death was dose dependently increased when GLS1 was inhibited by BPTA while inhibition of mitochondrial pyruvate carrier (MPC) attenuated cell death in NMDA-challenged OHC. Fumarate and malate were increased after IR 1h in CA2-4,DG and this was reversed by βIIV5-3 what correlated with GLS1 activity increases and earlier showed elevation of neuronal death (Krupska et al., 2017). The present study illustrates that CA2-4,DG resistance to ischemic episode at least partially rely on glutamine and glutamate utilization in mitochondria as a source of carbon to tricarboxylic acid cycle. This phenomenon depends on modulation of GLS1 activity by PKCβII and remodeling of MPC: all these do not occur in ischemia-vulnerable CA1.

## 1. Introduction

The brain has developed several endogenous, adaptive mechanisms (called endogenous neuroprotection) to protect itself from the harmful consequences of ischemia/reperfusion (IR) injury. Understanding of such phenomena may be important for the development of new neuroprotective strategies. An example of such mechanism is the increased resistance to IR injury of the abdominal region of the hippocampus (CA2-4,DG) versus the dorsal, ischemia-vulnerable region (CA1) [1,2,3]. Searching for mechanisms of CA2-4,DG resistance we previously showed that PKCβII translocation to mitochondria may be involved [4]. Mitochondria are key organelles for brain health. The high demand of energy used by glutamatergic neurons to maintain activity of energy-expensive ion pumps and regulate neurotransmitter release and uptake [5] is covered by ATP mostly produced by the oxidative phosphorylation of ADP. This process is driven by energy released during oxidation of reductive equivalents (NADH and FADH_2_) in the respiratory chain. In neurons reductive equivalents are delivered mostly but not exclusively by tricarboxylic acid cycle (TCA). Therefore, continuous delivery, maintaining at the sufficient cellular concentration and a proper metabolism of substrates which may be used as intermediates of TCA is of crucial significance. In fact, a growing number of studies indicate the participation of anaplerotic reactions in the functioning and survival of neurons under stress [6,7]. It was shown that glutamine and glutamate are the main players not only in neurotransmission but above all are key metabolites in brain bioenergetics. In mitochondria, glutamine is converted to glutamate and ammonia by phosphate-activated mitochondrial glutaminase (GLS1) [8]. Next, glutamate is converted to 2-oxoglutarate by two types of reactions. In one, the amino group of glutamate is transferred to an oxoacid, producing 2-oxoglutarate and appropriate amino acid. This reaction is catalyzed by aminotransferases. In the second one, glutamate undergoes oxidative deamination catalyzed by glutamate dehydrogenase (GDH), releasing ammonia and producing 2-oxoglutarate. GLS1 is suggested to be linked with elevated metabolism of microglia [9] and decreased reactive oxygen species (ROS) level in both normal and cancer cells [10]. Basically, GLS1 mediates conversion of glutamine to glutamate, supporting excitatory neurotransmission in the mammalian central nervous system. After uptake by astrocytes glutamate is converted to glutamine with participation of glutamine synthetase. Glutamine in turn is released to the extracellular space and taken up by neighboring neurons. There, the phosphate-activated glutaminase catalyzes re-conversion of glutamine to glutamate and ammonia. Glutamate is stored in the synaptic vesicles and released to the synaptic cleft upon cell excitation. Both neurons and glial cells contain two isoforms of glutaminase called kidney-type glutaminase (KGA) and glutaminase C (GAC). These two splice variants of *gls1* gene catalyze the same reactions. In neurons glutamate is mainly released as a neurotransmitter but it is also converted to 2-oxoglutarate by glutamate dehydrogenase (GDH) [6] and by glutamate oxaloacetate transaminase (GOT) [11]. Therefore, glutamate is an important respiratory substrate maintaining TCA cycle and cell survival upon impaired mitochondrial pyruvate transport [6]. The mitochondrial pyruvate carrier (MPC) genes, *mpc1* and *mpc2*, were identified to express proteins which form a transporter complex to control rate-limiting pyruvate transportation through the inner mitochondrial membrane [12,13]. Pyruvate uptake is mediated by a heterocomplex formed by MPC2 and MPC1 [12]. Deficiencies in MPC function reduce pyruvate entry into mitochondria and then the TCA cycle, leading to an elevated compensatory usage of glutamate [6,14]. Moreover, suppression of pyruvate transport induces the use of lipids and amino acids as catabolic and anabolic substrates [14]. It also was shown that the defective mitochondrial pyruvate flux alters neuronal function [15] while some reports indicate neuroprotective effect of MPC inhibition [16].

Mitochondrial enzymes are modulated by several signaling pathways which have different effects on their activity. It was reported that N-terminal phosphorylation of glutaminase C (GAC) at serine 95 decreases its enzymatic activity and cancer cell migration [17] while phosphorylation on GAC’s serine 314 catalyzed by PKCε leads to protein activation and an elevation of glutaminase activity and tumor malignancy [18]. Protein kinase C epsilon was shown to positively modulate respiration of synaptic mitochondria and restores mitochondrial activity of GOT2 leading to postischemic neuroprotection [19,20].

Earlier we showed that PKCβII translocates to postischemic mitochondria in ischemia-resistant CA2-4,DG sector and selective inhibition of this enzyme by βIIV5-3 peptide resulted in increased postischemic neuronal death in all hippocampal regions [4].

Here we have continued previously published studies on the role of this protein kinase in mitochondria and evaluated phosphate-activated mitochondrial glutaminase (GLS1) as one of PKCβII-interacting proteins. We have tested the hypothesis that the PKCβII interacts with GLS1 and by this mean may have a neuroprotective function in ischemia-resistant CA2-4,DG.

We suggest that metabolic plasticity of CA2-4,DG at least partially based on the GLS1 activity regulated by PKCβII and mitochondrial glutamate oxidation in concert with down regulation of MPC can facilitate an adaptation of CA2-4,DG region of the hippocampus to stressful conditions and minimizes effects of excitotoxic injury [21].

## 2. Results

### 2.1. PKCβII and GLS1 Glutaminase Isoform GAC but Not KGA Co-Localize in the Postischemic Mitochondria In Vivo

**Figure 1:**
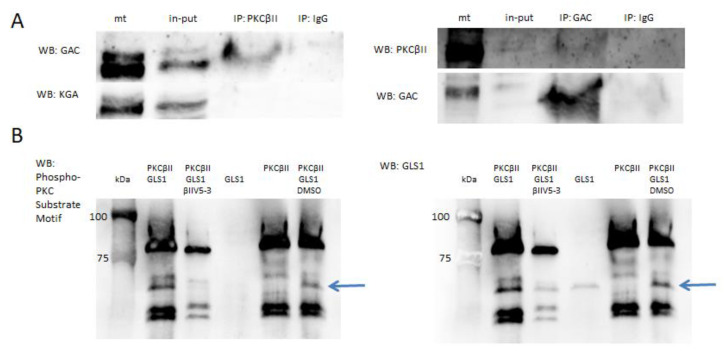
PKCβII and GLS1 glutaminase isoform GAC but not KGA co-localize in postischemic mitochondria in vivo. (**A**) Reciprocal co-immunoprecipitation of PKCβII and GAC shows co-localization of these two proteins in mitochondria isolated from CA2-4,DG after IR 1 h. (**B**) PKCβII is able to phosphorylate GLS1 in vitro, as showed by WB using anti-phosphorylated substrates of PKC (arrow), as described in Materials and Methods. Specific PKCβII inhibitor (50 µM βIIV5-3) reduced the extend of phosphorylation. DMSO (βIIV5-3 vehicle) did not influenced the reaction. The immunoblots are representative of four independent experiments. Mt—pure mitochondria, in-put—pure mitochondrial lysate for immunoprecipitation.

Here, we have demonstrated using a number of complementary methods that PKCβII interacts with isoform GAC of GLS1 in hippocampal regions CA2-4,DG after transient brain ischemia and 1 h reperfusion. First, using pure mitochondria isolated from the postischemic hippocampus we performed reciprocal co-immunoprecipitation. Western blot analysis of immunoprecipitates showed that PKCβII forms complexes with GAC but not with KGA isoform (Figure 1A). To further confirm the specificity of the complexes the pre-immune sera were used for immunoprecipitation, which did not instigate reactions with any of the proteins of interest. In control animals there was almost no PKCβII associated with mitochondria, thus we did not detect co-localization with GAC. Moreover, in vitro phosphorylation assay showed, that PKCβII was able to phosphorylate GLS1 and the extent of this reaction was reduced by specific PKCβII inhibitor (50 µM BIIV5-3) (Figure 1B).

### 2.2. Glutaminase Activity Is Higher in CA2-4,DG than in CA1 and Is Regulated by PKCβII

**Figure 2:**
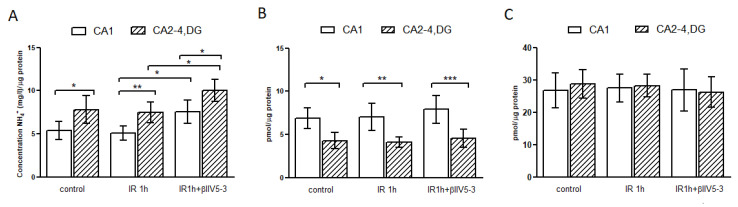
Glutaminase activity (GLS1) is higher in CA2-4,DG than in CA1. Mitochondria enriched fractions obtained from hippocampal CA1 and CA2-4,DG of control gerbils and gerbils subjected to 5 min ischemia and 1 h of reperfusion (IR 1h) with or without PKCβII inhibitor βIIV5-3 were used to measure glutaminase activity (**A**) and glutamine (**B**) and glutamate concentration (**C**). Results are mean ± SD (*n* = 4–7), * *p* < 0.05 ** *p* < 0.01 *** *p* < 0.001.

In these experiments the mitochondria-enriched fractions obtained from hippocampal CA1 and CA2-4,DG of control gerbils and gerbils subjected to 5 min ischemia and 1 h of reperfusion were examined. As demonstrated in Figure 2A, glutaminase activity was significantly higher in CA2-4,DG than in CA1 in control gerbils and remained unchanged after ischemia and reperfusion. Additionally, significantly increased activity of GLS1 was observed in samples obtained from animals subjected to ischemia and treated with PKCβII inhibitor βIIV5-3. The obtained results were confirmed by measuring glutamine and glutamate concentration. The glutamine levels were significantly lower in CA2-4,DG than in CA1 in control and IR 1h that confirms higher GLS1 activity in this region (Figure 2B). The glutamate levels were the same irrespectively of the experimental group and studied hippocampal region (Figure 2C).

### 2.3. GAC, One of GLS1 Isoforms, Is Responsible for Higher GLS1 Activity in CA2-4,DG

**Figure 3:**
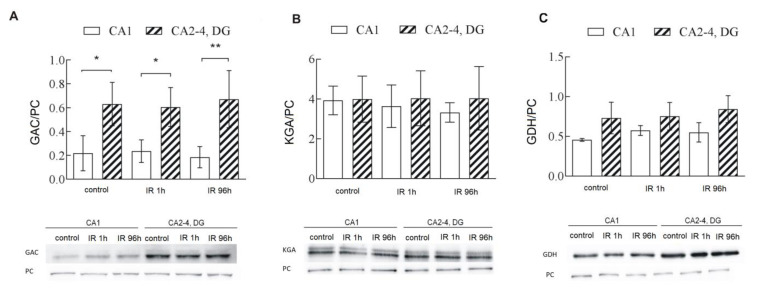
Mitochondrial level of GLS1 isoforms: GAC (**A**) and KGA (**B**) and glutamate dehydrogenase (GDH) (**C**) in controls and gerbils subjected to 5 min ischemia and 1 and 96 h of reperfusion (IR 1 h, IR 96 h). 20 µg of pure mitochondrial fractions were separated by 10% SDS-PAGE and analyzed by Western blot with anti-GAC, anti-KGA, or anti-GDH and anti-pyruvate carboxylase (PC) to assess the gel loading. The immunoblots are representative of four independent experiments. Densities of GAC, KGA and GDH bands were evaluated, and data are expressed as a percentage of PC (mean ± SD, *n* = 4). * *p* < 0.05, ** *p* < 0.001.

Mitochondrial GAC immunoreactivity in the control tissue was found to be significantly greater in CA2-4,DG than in CA1 and this difference persisted despite IR (Figure 3A) while, mitochondrial KGA immunoreactivity remained unchanged after IR and was the same in both hippocampal regions (Figure 3B). Based on these results, we can speculate that the higher GLS1 activity in CA2-4,DG is a consequence of the higher GAC level in this region. Moreover, glutamate dehydrogenase (GDH) immunoreactivity in control tissue was found significantly greater in CA2-4,DG than in CA1 and this difference persisted despite IR (Figure 3C). This may indicate a higher GDH activity in CA2-4,DG and promotion of 2-oxoglutarate formation.

### 2.4. Glutaminase and Mitochondrial Pyruvate Carrier Contribute to the Survival Rate of Hippocampal Slices

**Figure 4:**
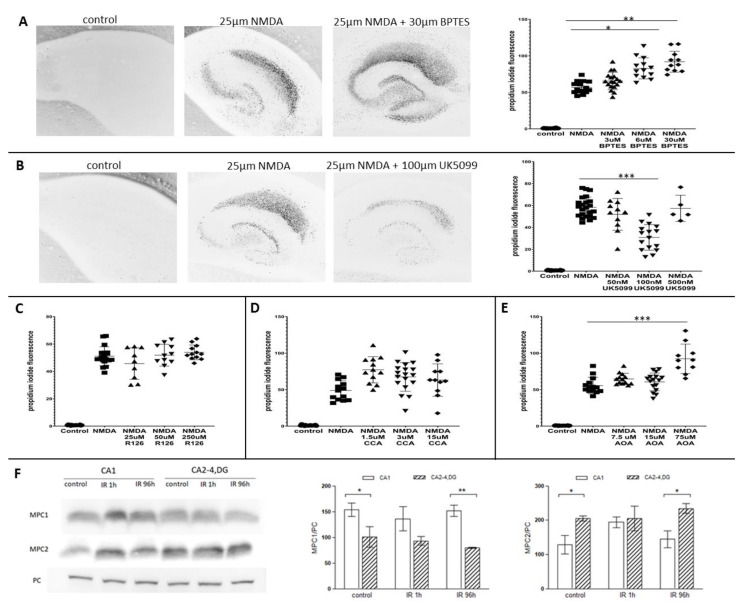
Glutaminase and mitochondrial pyruvate carrier activity contribute to the survival rate of hippocampal organotypic culture (OHC). (**A**) Confocal microscopy images of hippocampal slices in OHC and scatter plot graph showing the ratio between death cells in controls, slices treated with 25 μM NMDA or slices treated with NMDA and GLS1 inhibitor (BPTES). (**B**) Confocal microscopy images of hippocampal slices in OHC and scatter plot graph showing the ratio between death cells in controls, slices treated with 25 μM NMDA or slices treated with NMDA and MPC inhibitor (UK5099). (**C**–**E**) Scatter plot graphs showing the ratio between death cells in controls, slices treated with NMDA or slices treated with NMDA and R126 (inhibitor of glutamate dehydrogenase), CCA (inhibitor of monocarboxylic acid transport, including lactate and pyruvate transport) and AOA (an inhibitor of transaminases), respectively. *n* = 3 independent experiments for each condition, * *p* < 0.05, ** *p* < 0.01, *** *p* < 0.001. (**F**) Amount of mitochondrial pyruvate carrier subunits (MPC1 and MPC2) in control and gerbils subjected to 5 min ischemia and reperfusion (IR). 25 µg of cell extracts were separated by 15% SDS-PAGE and analyzed by Western blot with anti-MPC1, anti-MPC2 and anti-pyruvate carboxylase (PC) to assess the gel loading. The immunoblots are representative of four independent experiments. Densities of MPCs bands were evaluated and data are expressed as a percentage of PC (mean ± SD, *n* = 4). * *p* < 0.05, ** *p* < 0.01.

To further investigate possible fate of glutamate in mitochondria, organotypic hippocampal slices were used to test cell viability as a consequence of inhibition of glutaminase GLS1 (BPTES), glutamate dehydrogenase 1 (R162), mitochondrial pyruvate carrier (UK-5099), transaminases (AOA) or monocarboxylic acid transport, including lactate and pyruvate transport (CCA) upon NMDAR activation. None of the molecules used affected the viability of control neurons. As shown in Figure 4A the inhibition of GLS1 dose dependently and significantly increased cell death. Conversely, inhibition of mitochondrial pyruvate carrier (MPC) activity increased cell viability (Figure 4B). Inhibition of GDH or monocarboxylic acid transporters did not influence cell survival after NMDA challenge (Figure 4C,D, respectively); however, inhibition of aminotransferases by AOA seemed to enhance cell death in OHC (Figure 4E). Taken together, these results suggest that reducing the availability of pyruvate for TCA may increase the use of glutamate as an energy source under NMDA excitotoxicity and GLS1 activity.

Additionally, Western blot analysis of MPC1 and MPC2 in gerbil hippocampal sectors in time course after transient ischemia showed a different subunit composition of mitochondrial pyruvate carrier (Figure 4F). Under control conditions, the proportions between the subunits in the tested sectors showed differences in the level of both MPC1 and MPC2. In ischemia-resistant sector, immunoreactivity of MPC2 was higher than in CA1 and unchanged in the time course of the experiment while in CA1 after IR 1h, the amount of MPC2 significantly but transiently increased. In contrast, MPC1 level was significantly higher in ischemia-vulnerable CA1 then in CA2-4,DG in control conditions. After ischemic insult, the immunoreactivity of MPC1 in CA2-4,DG had a tendency to decrease and remained at a lower level for a longer time of reperfusion while in CA1 there was a tendency to increase after IR1h. Such a different MCP subunits composition and reaction on IR in hippocampal parts that are differently sensitive to this insult might suggest various changes in activity of MPC after ischemic episode.

### 2.5. Metabolite Analysis

**Figure 5:**
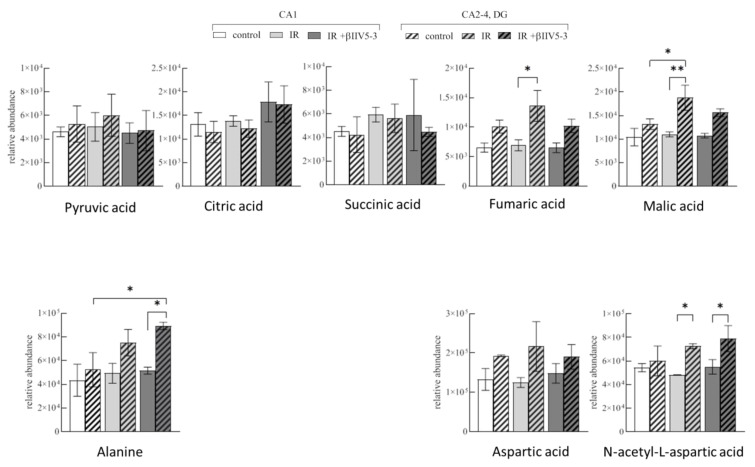
Relative abundance of pyruvate, alanine, N-acetyl-L-aspartate and TCA cycle intermediates in mitochondria isolated from hippocampal CA1 and CA2-4,DG of control and gerbils subjected to IR 1h treated or untreated with PKCβII inhibitor βIIV5-3 peptide as measured by gas chromatography-mass spectrometry. * *p* < 0.05, ** *p* < 0.001. Data are shown as mean ± SD, *n* = 3.

The principal component analysis (PCA-X) scores plot of the pre-processed GC-MS data displayed the tight clustering of QCs, indicating precision, reliability, and reproducibility of the analysis quality (Supplementary Figure S1). Furthermore, a clear separation was observed according to the PC1 axis of the samples based on their corresponding hippocampal structure origin, CA1 or CA2-4,DG. Moreover, a supervised PLS-DA analysis performed for the CA1 and CA2-4,DG model revealed the separation of the control, IR 1h and IR 1h + βIIV5-3 samples (Supplementary Figure S2). To identify the most significant metabolites contributing to the specified interpretation, CA1 vs. CA2-4,DG the average relative abundance was evaluated. Metabolomic analyses revealed that the concentration of TCA cycle intermediates fumarate and malate in CA2-4,DG were significantly elevated after IR 1h (Figure 5), whereas pyruvate, citrate, and succinate were unchanged in this section of the hippocampus. Moreover, the inhibition of PKCβII reverses ischemia-induced changes in fumarate and malate observed in ischemia resistant CA2-4,DG and induces formation of alanine in CA2-4,DG, that may reduce availability of pyruvate for TCA. Moreover, the concentration of N-acetyl-L-aspartate was significantly elevated after IR 1h, which may suggest a partial use of glutamate through transamination. Taken together, these results suggest that CA2-4,DG is naturally endowed with the possibility of a metabolic shift towards anaplerosis and this is modulated by PKCβII.

## 3. Discussion

The phenomenon of different sensitivity of brain regions to stress episodes has been known for years, but the mechanism of local resistance including relatively low susceptibility of CA2-4,DG to the IR stress is not fully explained. One of the studied threads that is gaining more and more experimental evidence is a concept of a metabolic plasticity of neurons. Glucose is an obligatory substrate in the brain but there are convincing data showing that neurons just like peripheral tissues, can use other, non-sugar substrates as an energy source. Beside acetyl-CoA derivatives (ketone bodies and fatty acids) glutamate seems to be the most principal among them [21].

The data presented here indicate that the relative resistance of CA2-4,DG to IR stress can be based on an elevated consumption of glutamine which is delivered by anaplerotic reactions as a carbon source for TCA cycle. Glutamate produced by phosphate-activated mitochondrial glutaminase (GLS1) from glutamine is converted to 2-oxoglutarate by glutamate dehydrogenase (GDH) which is present at a high concentration in mitochondria. It catalyzes the oxidative deamination of glutamate to 2-oxoglutarate and NH_3_. GDH is an allosteric protein modulated positively by ADP, GDP, and some amino acids and negatively by ATP, GTP, and NADH. NAD+/NADH ratio has important role in maintaining mitochondrial function against oxidative and ischemic stress [22,23]. As presented by Shijno and colleges (1998) the mitochondrial redox state in gerbil hippocampus measured at various time points after 5 min of forebrain ischemia showed increased NADH signal in all hippocampal areas, but reduction in mitochondrial redox ratio was greater and persistent in CA1 than in other areas of the hippocampus [24]. This suggests possible long lasting inhibition of GDH in CA1 but not in ischemia resistant CA2-4,DG.

As we showed earlier, PKCβII is enriched in postischemic CA2-4,DG mitochondria where its exerts neuroprotective role [4]. As it has been shown here, the GAC isoform of mitochondrial glutaminases is one of mitochondrial proteins interacting with this kinase. Moreover, more GAC is detected in CA2-4,DG than in the ischemia-sensitive CA1. In cancer cells, Ser95 and Ser314 residues of GAC were shown to undergo inhibitory and stimulatory phosphorylation, respectively [17,18]. Moreover, we have shown that PKCβII also can phosphorylate GLS1, and an inhibition of PKCβII results in the elevation of GLS1 activity in gerbil model of brain ischemia-reperfusion. The importance of maintaining proper activity of GLS1 in stressed neurons is also supported by our in vitro data. GLS1 inhibitor BAPTES, administered upon NMDA exposition, at the concentration of IC50 = 6 µM, strengthened neuronal damage, but it did not affect the survival rate of control cells. As demonstrated in Figure 2A, glutaminase activity was significantly higher in CA2-4,DG than in CA1 in control gerbils and remained unchanged after ischemia and reperfusion. GLS1 activation was observed after PKCβII inhibition in IR gerbil. Therefore, we suggest that both over activation of GLS1 (PKCβII inhibitor in vivo) and inhibition of activity in vitro are harmful to cells under IR stress. Thus, we propose that the neuron-protective mechanism of PKCβII in mitochondria involves phosphorylation and therefore modulation/downregulation of glutaminase activity. By this means generation of glutamate is substantially limited. Moreover, reduced availability of pyruvate for mitochondrial oxidation supports more effective oxidation of glutamate. This concept is additionally supported by data showing that an inhibition of the mitochondrial pyruvate carrier leads to activation of GDH and redirecting glutamine/glutamate metabolism to supply TCA cycle with 5-carbon metabolite [6]. Moreover, as it is shown here, MPC inhibition in hippocampal slices upon NMDA challenge in vitro protects neurons. Similarly, the tendency to postischemic reduction of MPC1 protein in CA2-4,DG and oppositely, elevation of MPC2 protein in CA1 in vivo suggest that mitochondrial pyruvate carrier regulates/limits/controls a transport and following conversion of cytosolic pyruvate to mitochondrial acetyl-CoA thus its use in TCA cycle in the IR-resistant part of the hippocampus. This regulation has not been found in CA1, which strengthens the hypothesis about the participation of MPC, GLS1, PKCβII, and glutamate in CA2-4,DG resistance in excitotoxic stress. Moreover, MPC1 seems to be a key target for UK-5099 [25], which is an inhibitor of mitochondrial pyruvate carrier and showed protection in hippocampal slices in vitro. So together, we can speculate that low MPC1 amount in gerbil CA2-4,DG and its further postischemic reduction may suggest lower activity of pyruvate carrier in vivo. Our hypothesis is also supported by previously published facts. There are data presented by Bricker et al. (2012) showing an elevation in fumarate and malate as a consequence of MPC1 deficiency in *Drosophila* grown on standard medium which allowed mutants defective in carbohydrate metabolism to correct their life span [26]. On the other hand, it was shown that an inhibition of the mitochondrial pyruvate carrier protects from excitotoxic neuronal death suggesting neuronal metabolic flexibility and increase in reliance on glutamate as a fuel [21]. It was also shown that decreased expression of MPC1, interfered with pyruvate entry into mitochondria and increased cellular reliance on glutamine oxidation and the pentose phosphate pathway to maintain reduced NAD phosphate (NADPH) homeostasis in breast cancer [27]. Finally, one can conclude that the unequal susceptibility of hippocampal regions relies on differences in a preferential utilization of glutamate or pyruvate as respiratory substrates. Despite the fact that the cellular level of both compounds are similar and are not changed upon IR, efficient oxidation of glutamate protects CA2-4,DG region from glutamate excitotoxicity. In contrast, preferential oxidation of pyruvate in the CA1 region reduces oxidative metabolism of glutamate in TCA, so potentially increases a pool of glutamate which may be used as the neurotransmitter. More effective mitochondrial pyruvate usage in CA1 is in line with these processes.

In our experiments, the inhibition of PKCβII resulted in an activation of GLS1 in both hippocampal regions and reversed (prevented) ischemia-induced changes in the cellular level of fumarate and malate and induced formation of alanine in CA2-4,DG. This may reduce pyruvate oxidation and further enhance neuron death due to limited availability of acetyl-CoA. Additionally, significantly elevated concentration of N-acetyl-L-aspartate (NNA) after IR 1h and even more after PKCβII inhibition, may suggest an elevated use of glutamate by transamination reaction with 2-oxaloacetate catalyzed by ASPAT. Our in vitro data showed an acceleration of cell death in the presence of AOA, transaminase inhibitor, that is in line with other data where an elevation of neuronal death was observed after OGD when GOT2 activity was reduced [19]. Restoring aminotransferase activity by PKCε specific activator revealed novel protective target and mechanisms against ischemic injury [19]. On the other hand, there is no consensus on N-acetyl-L-aspartate’s principle metabolic or neurochemical functions, but high levels of brain N-acetyl-L-aspartate were found in many Canavan patients, suggesting that excess NAA may have toxic effects in the CNS [28]. The above considerations probably do not explain all aspects of the selective resistance of a particular part of the hippocampus. However, they focus on the metabolic aspect of this response. On the other hand, a complete map of metabolic processes involved is much more complicated and contains more elements including changes in ammonia concentration and redox potential which are closely related to glutamate metabolism. It is worth highlighting that a mechanism of protection against oxygen glucose deprivation based on accelerated glutamate consumption by transamination followed by oxidation was postulated previously by Xu and coworkers, 2020 [19]. Thou, we suggest: (i) PKCβII entry into mitochondria upon IR stress is substantially greater in the abdominal part of hippocampus [4]. This results in an inhibition/downregulation of mitochondrial glutaminase thus reduces glutamate formation. In CA1 such a mechanism has not been found. (ii) In the CA2-4,DG of hippocampus IR stress reduces pyruvate entry into mitochondria and therefore makes glutamate to be more intensively oxidized. In the CA1 region stronger competition between pyruvate and glutamate oxidation reduces glutamate consumption. Thus, limited glutamate supply and a switch from pyruvate to glutamate oxidation seem to be behind CA2-4,DG ability to survive glutamatergic excitotoxicity in vitro and IR stress in vivo; CA1 region of the hippocampus does not have such a possibility.

## 4. Materials and Methods

### 4.1. Ethical Statement and Animals

All experimental procedures were approved by the Local Ethics Committee for Animal Experimentation and every effort was made to minimize animal suffering. Wistar rats (7-day-old pups) and Mongolian gerbils (*Meriones unguiculatus*) were obtained from the Animal House of the Mossakowski Medical Research Institute of the Polish Academy of Sciences.

### 4.2. Transient Brain Ischemia in Gerbils

Gerbils weighing 60–70 g were subjected to transient brain ischemia by means of a 5 min bilateral ligation of common carotid arteries under isoflurane anesthesia, in strictly controlled normothermic conditions as described previously [4,29]. For selected experiments, βIIV5-3 peptide (3 mg/kg) was dissolved in saline and injected directly to the left carotid artery in the course of the ischemic insult as described earlier [4]. Following ischemia, the animals recovered for 1 or 96 h prior to decapitation, with the CA1 and CA2-4,DG regions of the hippocampus then being isolated for protein. Hippocampi from non-treated animals served as controls.

### 4.3. Sample Preparation and Isolation of Pure Mitochondria

A particulate and soluble fractions together with a pure-mitochondria fraction were obtained from the CA1 and CA2-4,DG regions of the hippocampi of control and ischemic gerbils. Hippocampi were homogenized in ice-cold isotonic buffer (15 mM Tris/HCl, pH 7.5, 0.25 M sucrose, 1 mM MgCl_2_, 1 mM EGTA, 2 mM EDTA, 1 mM PMSF, and 1 mM DTT), prior to centrifugation (1000× *g*, 10 min, 4 °C). The supernatant was centrifuged at 11,000× *g* for 20 min at 4 °C to yield a particulate fraction enriched with mitochondria (P2). The pure mitochondrial pellet was obtained after centrifugation of P2 (100,000× *g*, 30 min, 4 °C) with 12% Ficoll, as described earlier [4,30]. For Western blot analysis of MPC1 and MPC2 the tissue was homogenized under nondenaturing conditions, in cell lysis buffer containing 20 mM Tris–HCl at pH 7.5, 150 mM NaCl, 1 mM Na_2_EDTA, 1 mM EGTA, 1% Triton, 2.5 mM sodium pyrophosphate, 1 mM β-glycerophosphate, 1 mM Na_3_VO_4_, 1 μg/mL leupeptin, and 1 mM PMSF (Cell Signaling Technology, Danvers, MA, USA). After homogenization samples were sonicated four times for 5 s and centrifuged 14,000× *g* at 4 °C for 20 min to obtain clear tissue lysates. The protein concentration was determined using a Modified Lowry Protein Assay Kit (Thermo Scientific, Grand Island, NY, USA).

### 4.4. Co-Immunoprecipitations

The mitochondria (200 µg), obtained from CA2-4,DG hippocampi of gerbils subjected to 5 min ischemia and 1 h reperfusion, were lysed in 500 μL of lysis buffer: 10 mM Tris-HCl pH 7.5, 150 mM NaCl, 1% Triton X-100, 0.5% NP-40, 1mM PMSF. Samples were cleared of insoluble debris by centrifugation at 10,000× *g* for 20 min and preincubated with 20 µL protein A-Sepharose beads (Sigma-Aldrich, Poznan, Poland) to remove proteins nonspecifically associated with the beads. Resulting supernatant was incubated with anti-PKCβII (Santa Cruz Biotechnology, Dallas, TX, USA, Sc-13149)) or anti-GAC (Proteintech, Manchester, UK, 19958-1-AP) or anti-KGA (Proteintech, Manchester, UK, 20170-1-AP) antibodies conjugated to protein A beads according to the manufacturer protocol (Seize X Protein A Immunoprecipitation Kit, Pierce, Dallas, TX, USA). Proteins associated with antibodies were eluted with the electrophoresis sample buffer, resolved by 10% polyacrylamide gel electrophoresis and analyzed by reciprocal Western blots.

### 4.5. GLS1 Phosphorylation by PKCβII in In Vitro Assay

Recombinant proteins: 200 ng PKCβII (Sigma-Aldrich, Poznan, Poland, P3287) and 200 ng GLS1 (ORIGENE, Herford, Germany, TP306265) were mixed with 1x kinase buffer (Cell Signaling Technology, Danvers, MA, USA, 9802S) in the presence or without PKCβII inhibitor 50µM βIIV5-3 [4,31] dissolved in 5% DMSO. Control samples contained PKCβII or GLS1 only. The reactions were initiated by the addition of 10 mM ATP (Cell Signaling Technology, Danvers, MA, USA, 9804S) in 1x kinase buffer (final 200 μM ATP) and run for 20 min at 30 °C with agitation (300 rpm). The reactions were stopped with 5× Laemmli buffer. Samples were electrophoresed on 10% SDS-PAGE gel and then Western blot was performed using anti-Phospho-PKC Substrate Motif [(R/K)XpSX(R/K)] MultiMab™ Rabbit mAb mix (Cell Signaling Technology, Danvers, MA, USA, 6967) and anti- GLS1 (Proteintech, Manchester, UK, 12855-1-AP).

### 4.6. Glutaminase Activity Measurement

Glutaminase activity was measured by the increase in ammonium ions according to the method described by Romero-Gomez with slight modifications [32]. Briefly, 25 μL of mitochondria-enriched fraction (25 μg) were added to 25 μL of reaction medium (150 mM K_2_HPO_4_, 171 mM L- glutamine, 1 mM NH_4_Cl at pH 8.0). Blanks were prepared as followed with addition of 10 μL of 10% trichloroacetic acid (TCA). After 2 h incubation in 37 °C the reaction was stopped with 10% TCA, incubated (15 min on ice) and centrifuged (12,000× *g*, 5 min, 4 °C). The obtained supernatants (20 μL) were combined with 130 μL of OPA based reagent (0.2 M K_2_HPO_4_, pH 7.4, 56 mL/l ethanol, 10 mM O-phthaldialdehyde, 0.4 mM β-mercaptoethanol) and incubated in dark at room temperature for 35 min. Absorbance was measured at 405 nm. Standard curve of NH_4_Cl (10–700 mg/L) was prepared for calculations of the results.

### 4.7. Glutamine and Glutamate Concentration Measurement

Glutamine and glutamate concentration were analyzed in mitochondria-enriched fraction using high-performance liquid chromatography (HPLC) with fluorescence detection with O-phthaldialdehyde as described earlier [33].

### 4.8. Western Blot Analysis

The mitochondrial fractions or the particulate fractions (20 µg) were separated by SDS-PAGE, transferred to nitrocellulose membrane (Amersham Protran, GE Healthcare, Sigma-Aldrich, Poznań, Poland) and analyzed by Western blot using rabbit polyclonal anti-GAC (Proteintech, Manchester, UK, 19958-1-AP), anti-KGA (Proteintech, Manchester, UK, 20170-1-AP), anti-GDH (Proteintech, Manchester, UK, 14299-1-AP), MPC1 as well as MPC2 (Cell Signaling Technology, Danvers, MA, USA, (D2L9I) Rabbit mAb 14,462 and (D4I7G) Rabbit mAb 46141, respectively). Equal protein loading was confirmed by pyruvate carboxylase antibody (PC, Proteintech, Manchester, UK, 16588-1-AP). Protein bands were detected with horseradish peroxidase-coupled secondary antibody, and enhanced with chemiluminescent substrate (ECL Western Blotting Detection Reagents, GE Healthcare, Sigma-Aldrich, Poznań, Poland). The bands were detected and evaluated by densitometry and normalized using FUSION FX (VILBER LOURMAT, Marne-la-Vallée, France) apparatus and software.

### 4.9. Organotypic Hippocampal Slice Culture

Organotypic hippocampal slices were used to examine the effect of N-methyl-D-aspartic acid (NMDA) and inhibition of selected mitochondrial proteins on neuronal death. The slices were prepared from 7-day old Wistar rats, after Stoppini, though with slight modifications [4,34]. Slices 400 µm thick were cultured in medium containing 50% Neurobasal (Gibco, Thermo Fisher Scientific, Grand Island, NY, USA), 25% horse serum (Gibco, Thermo Fisher Scientific, Grand Island, NY, USA), 20% HBSS (Gibco, Thermo Fisher Scientific, Grand Island, NY, USA), B27 supplement (Gibco, Thermo Fisher Scientific, Grand Island, NY, USA, 1:50 dilution), 1 M HEPES (Gibco, Thermo Fisher Scientific, Grand Island, NY, USA), 5 mg/mL glucose (Sigma-Aldrich, Poznań, Poland), 0.5 mM glutamax (Gibco, Thermo Fisher Scientific, Grand Island, NY, USA), and Antibiotic Antimycotic Solution (Sigma-Aldrich, Poznan, Poland, 1:100 dilution). Cultures were started in a 25% horse serum-containing medium which was gradually removed starting from DIV three through six. Cultures were maintained in a humidified atmosphere of air and 5% CO_2_, at 36 °C for 8 days. Neuronal death was induced by 25 µM NMDA (3 h in culture), which was added to the culture simultaneously with one of the inhibitors: glutaminase (BPTES, TOCRIS, Warsaw, Poland, 3–30 μM), mitochondrial pyruvate carrier (UK-5099, Sigma-Aldrich, Poznan, Poland, 50–500 nM), glutamate dehydrogenase (R162, TOCRIS, Warsaw, Poland, 25–250 μM), inhibitor of monocarboxylic acid transport, including lactate and pyruvate transport (α-Cyano-4-hydroxycinnamic acid (CCA), Sigma-Aldrich, Poznan, Poland, 1.5–15 μM) or transaminases (AOA, Sigma-Aldrich, Poznan, Poland, 7.5–75 μM). After 3 h of incubation, NMDA was removed while inhibitor was present in the culture medium until the end of the experiment. Quantification of cell death was performed 24 h after NMDA exposure by measuring the intensity of fluorescent cell death marker—propidium iodide (PI) using an Axiovert fluorescence microscope (Carl Zeiss AG, Jena, Germany). Values were normalized to maximal fluorescent intensity, obtained by treating slices with 100 µM NMDA. At least six slices were analyzed in each experiment under each condition.

### 4.10. Untargeted Metabolomics Analysis

Before metabolite extraction, the mitochondrial pellet was re-suspended in 80 µL of ultrapure water and the samples were lysed by four freeze-thaw cycles in liquid nitrogen. Subsequently, the samples were mixed with three-volume of cold acetonitrile (−20 °C) containing 0.125 mM 4-chlorophenylalanine (IS) and centrifuged (16000× *g*, 4 °C, 10 min). The resulting supernatant (150 µL) was transferred to gas chromatography (GC) vial and evaporated to dryness (SpeedvacConcentrator, Thermo Fisher Scientific, Waltham, MA, USA). The pellet was used in further analysis to estimate protein concentration. The two-step derivatization procedure was performed as previously described [35]. A GC system (7890A, Agilent Technologies, Waldbronn, Germany) coupled with a mass spectrometer with triple-Axis detector (5975C, Agilent Technologies, Waldbronn, Germany), was used for analysis, following previously described parameters [36]. Acquired data were processed applying spectral deconvolution and alignment. Compound identification was performed with the target metabolite Fiehn GC-MS Metabolomics RTL (Retention Time Locked) library (G1676AA, Agilent Technologies, Waldbronn, Germany), the in-house built CEMBIO-library and the NIST (National Institute of Standards and Technology, Gaithersburg, MD, USA) mass spectra library (Ver. 2014). Quality control and quality assurance procedures were applied according to published guidelines [37,38]. Acquired data were evaluated by examination of reproducibility of sample treatment procedure and analytical performance by raw data inspection (PCA-X, Principal Component Analysis). Instrumental variation detected was corrected by QC samples applying the support vector regression algorithm (QC-SVRC) [39]. Variation of the compound measurements was calculated for QCs and expressed as relative standard deviation (% RSD) with the cut-off value RSD >30%. The replicate measurements were summarized to average values to reduce the influence of noise in downstream data analysis. The metabolite abundances were normalized with respect to the protein concentration. QC-SVRC normalization was performed using MATLAB scripts (Matlab R2015, Mathworks) other calculations in Excel (Microcoft). Multivariate analysis was performed in SIMCA-P+16.0 software (Umetrics, Umea, Sweden). See Supplementary Information for full methodological details.

### 4.11. Statistics

The significance of differences among groups was calculated using one-way analysis of variance followed by Bonferroni’s Multiple Comparison Test. A value of *p* < 0.05 was considered significant.

## Data Availability

The data presented in this study are available in this article and Supplementary Material.

## Supplementary Materials

The following are available online at https://www.mdpi.com/article/10.3390/ijms22168504/s1.
