# Supplementary material for: Mitochondrial Metabolism behind Region-Specific Resistance to Ischemia-Reperfusion Injury in Gerbil Hippocampus. Role of PKCβII and Phosphate-Activated Glutaminase
Source: Int J Mol Sci. 2021 Aug 7;22(16):8504. doi: 10.3390/ijms22168504 (PMC8395184; doi:10.3390/ijms22168504)
Supplement: Supplementary file 1 [file ijms-22-08504-s001.zip › ijms-1303603-supple picture.pptx]

## Slide 1
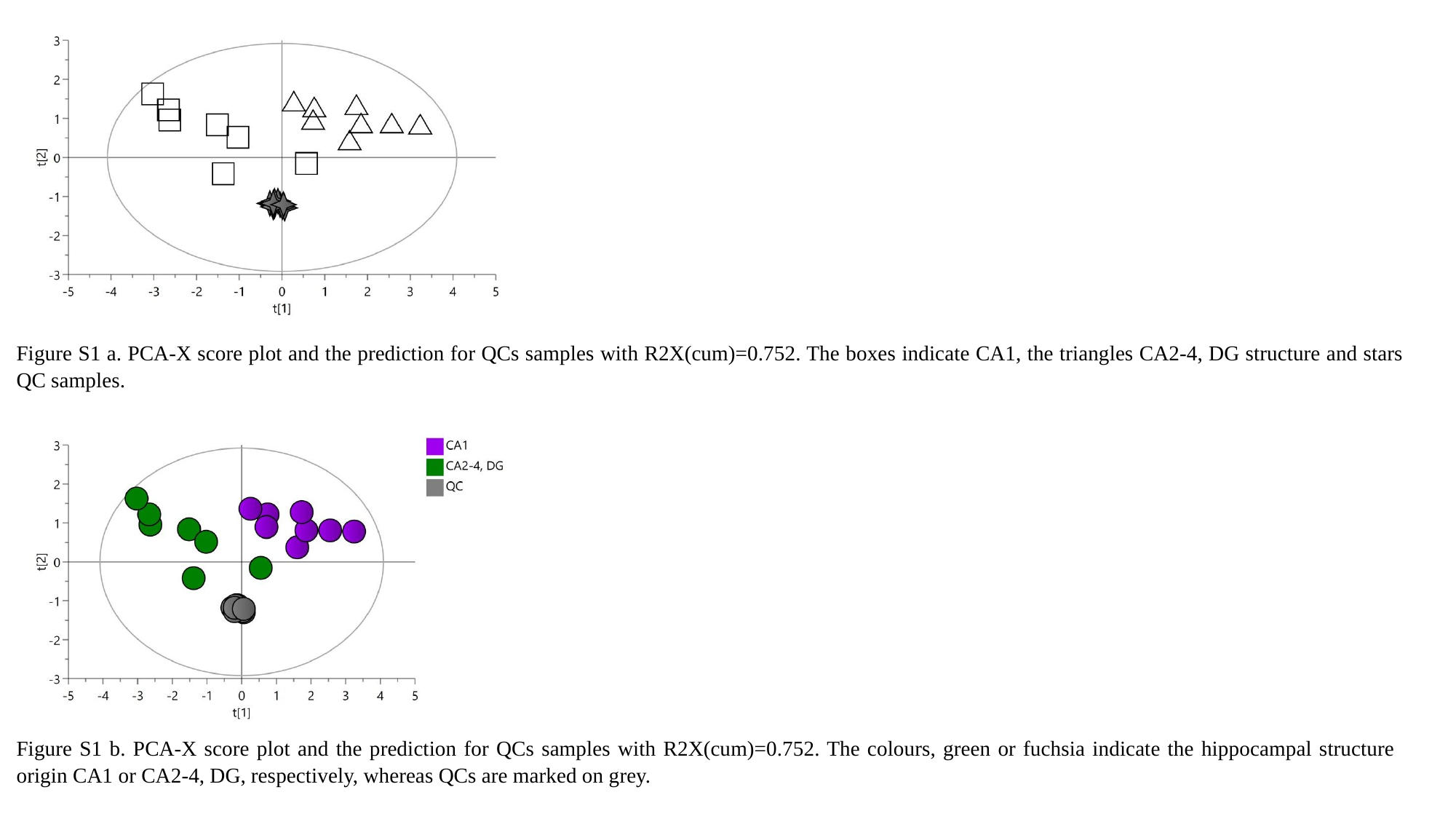

Figure S1 a. PCA-X score plot and the prediction for QCs samples with R2X(cum)=0.752. The boxes indicate CA1, the triangles CA2-4, DG structure and stars QC samples.
Figure S1 b. PCA-X score plot and the prediction for QCs samples with R2X(cum)=0.752. The colours, green or fuchsia indicate the hippocampal structure origin CA1 or CA2-4, DG, respectively, whereas QCs are marked on grey.

## Slide 2
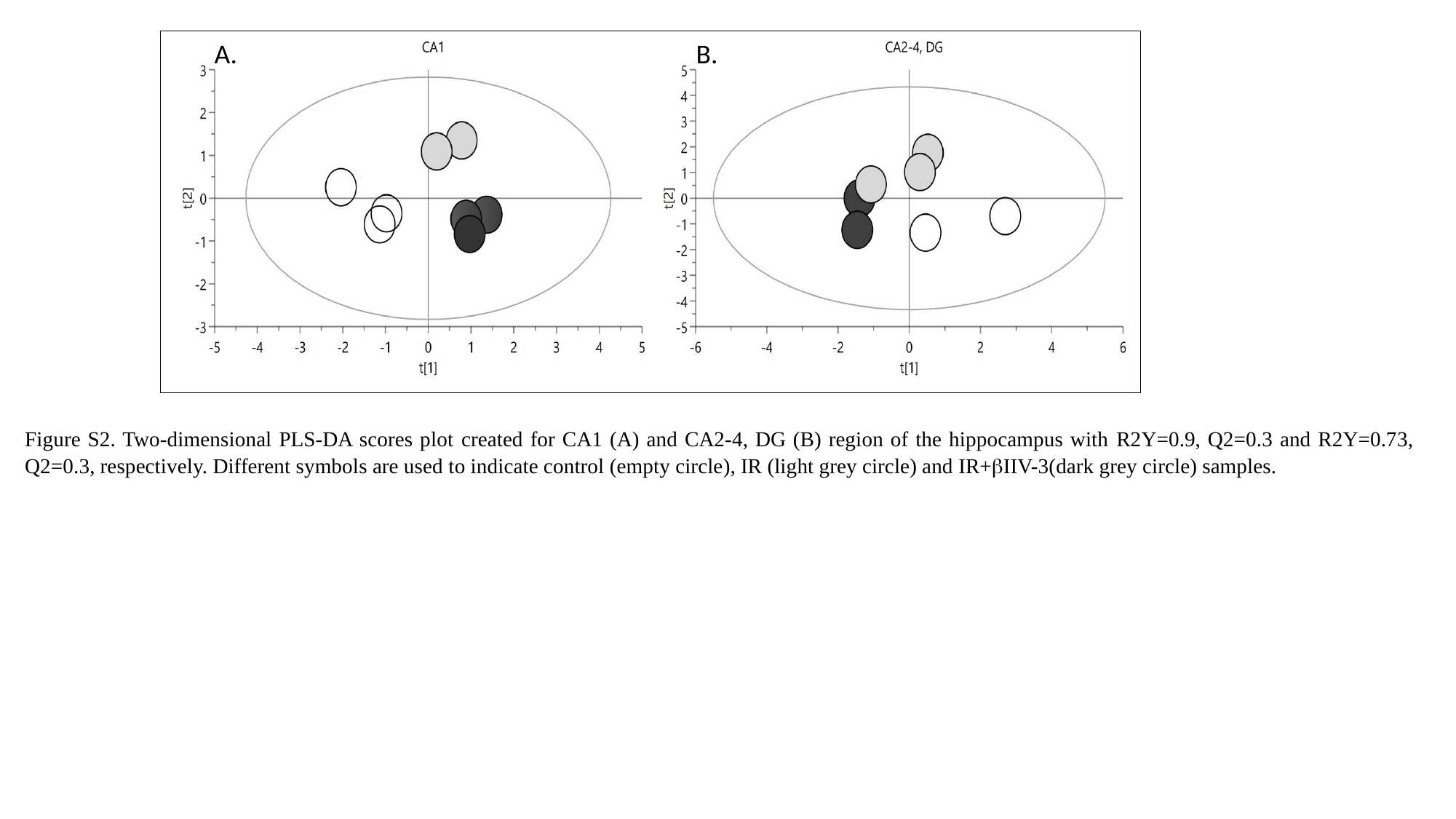

A.
B.
Figure S2. Two-dimensional PLS-DA scores plot created for CA1 (A) and CA2-4, DG (B) region of the hippocampus with R2Y=0.9, Q2=0.3 and R2Y=0.73, Q2=0.3, respectively. Different symbols are used to indicate control (empty circle), IR (light grey circle) and IR+βIIV-3(dark grey circle) samples.
