# Supplementary material for: Mitochondrial Metabolism behind Region-Specific Resistance to Ischemia-Reperfusion Injury in Gerbil Hippocampus. Role of PKCβII and Phosphate-Activated Glutaminase
Source: Int J Mol Sci. 2021 Aug 7;22(16):8504. doi: 10.3390/ijms22168504 (PMC8395184; doi:10.3390/ijms22168504)
Supplement: Supplementary file 1 [file ijms-22-08504-s001.zip › ijms-1303603-supple.pdf]

Untargeted Metabolomics analysis

Chemicals

Organic solvents (MS grade) acetonitrile, heptane, 2-propanol, O-Methoxyamine hydrochloride standard mix containing grain fatty acid methyl ester (FAME) mixture (C8:0–C22:1n9), C:18 methyl ester and 4-chlorophenylalanine were from Sigma-Aldrich (St. Louis, MO, USA). Sialylation-grade pyridine was from VWR International BHD Prolabo (Madrid, Spain). N,O-bis(trimethylsilyl)trifluoroacetamide with 1%trimethylchlorosilane was from Pierce Chemical Co (Rockford, IL, USA). Ultrapure water was generated by Milli-Qplus185 system (Millipore, Billerica, MA, USA).

Sample preparation

Before metabolite extraction, the mitochondrial pellet was re-suspended in 80 µL of ultrapure water and the samples were lysed by four freeze-thaw cycles in liquid nitrogen. Subsequently, the samples were mixed with three-volume of cold acetonitrile (-20 ℃) containing 0.125 mM 4-chlorophenylalanine (IS) and centrifuged (16000g, 4 ℃, 10 min). The resulting supernatant (150 µL) was transferred to GC vial with insert and then evaporated to dryness (SpeedvacConcentrator, Thermo Fisher Scientific, Waltham, MA, USA). The pellet was used in further analysis to estimate protein concentration. The two-step derivatization procedure was performed as previously described [1] consisting methoximation with O-methoxyamine hydrochloride (15 mg/mL) in pyridine, and silylation with N,O bis(trimethylsilyl)trifluoroacetamide (BSTFA) with 1% trimethylchlorosilane (TMCS). Finally, 50 µL of heptane was added to each vial, and the samples were vortex-mixed and centrifuged (2500 rpm, 20 ℃, 15 min) before GC-MS analysis.

Quality control (QC) samples were independently prepared by pooling equal volumes of each sample and following the same extraction procedure as applied for experimental samples. Analyte-free extraction blank sample was also considered [2].

Protein assay

The pellet was dissolved in 50 mM phosphoric acid (pH 8, adjusted with NaOH 1 M solution) and 1% SDS. The bicinchoninic acid (BCA) protein assay was performed according to the manufacturer’s protocol (Thermo Fisher Scientific, Rockford, IL, USA). To determine the accuracy of total protein measurement the analysis was performed in triplicate.

GC-Q-MS analysis

A GC system (7890A, Agilent Technologies, Waldbronn, Germany) coupled with a mass spectrometer with triple-Axis detector (5975C, Agilent Technologies, Waldbronn, Germany), was used for analysis. The derivatized sample (2 µL) was injected (split ratio 1:10, Restek 20782

deactivated glass-wool split liner) by an autosampler (7693, Agilent Technologies, Waldbronn, Germany) through a GC–Column DB5–MS (30 m length, 0.25 mm i.d. ,0.25 μm film 95% dimethyl/5% diphenylpolysiloxane) with an integrated precolumn (10 m J&W, Agilent). Carrier gas (He) flow rate through the column was set at 0.728 mL/min. The retention time locking (RTL) relative to the internal standard C18:0 methyl ester (RT at 19.66 min) was performed. The injector port was held at 250 ℃. The temperature gradient was programmed as follows: the initial oven temperature was set at 60 ℃ (held for 1 min) with a ramping rate of 10 ℃/min up to 325 ℃, maintained for10 min before cooling down with the total run time 37.5 min per sample. The detector transfer line, the filament source and the quadrupole temperature were set to 280 ℃, 230 ℃ and 150 ℃, respectively. MS detection was performed in electron impact (EI) mode at 70 eV. The mass spectrometer was operated in scan mode with a mass range of m/z 50–600 at a rate of 2.7 scan/s [3] .

A standard mix of fatty acid methyl esters (FAME C8-C30), extraction blank and QCs for system equilibration were injected at the beginning of the analytical batch, following QCs injections every 6 experimental samples, QC and blank injection at the end of the worklist. Each sample was analyzed in 3 repeated consecutive injections (technical replicates).

Data processing and compound identification

Raw data Total Ion Chromatograms (TIC) for data quality assessment was inspected with Agilent MassHunter Qualitative Analysis Ver. B.08.00 (Agilent Technologies, Santa Clara, CA, USA). Spectral deconvolution with Agilent Unknown Analysis tool (Ver. B.08.00. Agilent Technologies, Santa Clara, CA, USA) was used to extract the acquired data. Alignment of drift (by retention time and mass) and data filtering were performed with the Mass Profiler Professional ver. B.12.1 (Agilent Technologies, Santa Clara, CA, USA) software. Assignment of the target ion and the qualifiers, entire batch pre-processing and manual inspection of the data including peak area and RT integration was performed with Agilent MassHunter Quantitative Analysis (Ver. B.08.00, Agilent Technologies, Santa Clara, CA, USA). Compound identification was performed with the target metabolite Fiehn GC-MS Metabolomics RTL (Retention Time Locked) library (G1676AA, Agilent), the in-house built CEMBIO-library and the NIST (National Institute of Standards and Technology, Gaithersburg, MD, USA) mass spectra library (Ver. 2014). Raw data has been cleaned from unrelated features, contaminants originating from mitochondria extraction, purification or metabolite extraction procedure.

Data treatment

Quality control and quality assurance procedures were applied according to published guidelines [2,4]. Acquired data were evaluated by examination of reproducibility of sample treatment procedure and analytical performance by raw data inspection. Three analytical outliers belonging to the CA1 group: IR (n=1) and CA2-4, DG group: control (n=1) and IR+βIIV5-3 (n=1) were detected and excluded from further data analysis. Principal component analysis (PCA-X) was used to check for signal drift, variation in the signal measurement of QC samples, evaluation of replicate samples and potential outliers. Instrumental variation detected was corrected by QC samples applying the support vector regression algorithm (QC-SVRC) [5]. Variation of the compound measurements was calculated for QCs and expressed as relative standard deviation (%RSD) and the data with an RSD >30% in QC samples were not considered valid. The replicate measurements were summarized to average values to reduce the influence of noise in downstream data analysis. The metabolite abundances were normalised with respect to the protein concentration.

QC-SVRC normalization was performed using MATLAB scripts (Matlab R2015, Mathworks) other calculations in Excel (Microcoft). Multivariate analysis was performed in SIMCA-P+16.0 software (Umetrics, Umea, Sweden).

References

1. Garcia A, Barbas C (2011) Gas chromatography-mass spectrometry (GC-MS)-based metabolomics. Methods Mol Biol 708:191-204. doi:10.1007/978-1-61737-985-7_11
2. Dudzik D, Barbas-Bernardos C, Garcia A, Barbas C (2018) Quality assurance procedures for mass spectrometry untargeted metabolomics. a review. J Pharm Biomed Anal 147:149-173. doi:10.1016/j.jpba.2017.07.044
3. Gonzalez-Pena D, Dudzik D, Garcia A, Ancos B, Barbas C, Sanchez-Moreno C (2017) Metabolomic Fingerprinting in the Comprehensive Study of Liver Changes Associated with Onion Supplementation in Hypercholesterolemic Wistar Rats. Int J Mol Sci 18 (2). doi:10.3390/ijms18020267
4. Broadhurst D, Goodacre R, Reinke SN, Kuligowski J, Wilson ID, Lewis MR, Dunn WB (2018) Guidelines and considerations for the use of system suitability and quality control samples in mass spectrometry assays applied in untargeted clinical metabolomic studies. Metabolomics 14 (6). doi:ARTN 7210.1007/s11306-018-1367-3

5. Kuligowski J, Sanchez-Illana A, Sanjuan-Herraez D, Vento M, Quintas G (2015) Intra-batch effect correction in liquid chromatography-mass spectrometry using quality control samples and support vector regression (QC-SVRC). Analyst 140 (22):7810-7817. doi:10.1039/c5an01638j
